# Validation of the Schieman and Young measurement scales for work contact, work-family conflict, working conditions, psychological distress and sleep problems in construction industry professionals

**DOI:** 10.1186/s12889-018-6100-7

**Published:** 2018-10-24

**Authors:** Paul Bowen, Rajen Govender, Peter Edwards

**Affiliations:** 1 Department of Construction Economics and Management, University of Cape Town, Private Bag, Rondebosch, Cape Town, 7701 South Africa; 2 Violence, Injury and Peace Research Unit, South African Medical Research Council and Department of Sociology, University of Cape Town, Cape Town, South Africa; 3 School of Property, Construction and Project Management, RMIT University, Swanston Street, Melbourne, VIC Australia

**Keywords:** Schieman and Young (2013), Work contact, Work-family conflict, Psychological distress, Sleep problems, Measurement scales, Validation

## Abstract

**Background:**

This study examined the construct validity and internal consistency of modified versions of the job autonomy and control, job pressure, work contact, work-family conflict, psychological distress, and sleep problems scales developed by Schieman and Young (2013) among construction professionals through confirmatory factor analysis and tests of internal consistency.

**Methods:**

Using a cross-sectional design, survey data were collected from 942 South African construction professionals, of which 630 responses were considered for analysis. Confirmatory factor analysis was used to examine construct validity. Cronbach’s coefficient alpha was used to determine the internal consistency, and convergent validity was tested using correlation analysis.

**Results:**

The final CFA indicated very good model fit to the data (χ2 /*df* ratio = 2.11, IFI = .95, CFI = .95, RMSEA = .06, and Hoelter (95%) = 176). The scales demonstrated satisfactory internal consistency: .82; .91; .83; .90; .90; and .73, respectively. Convergent validity was largely demonstrated with respect to direction of association, but not in relation to magnitude. A limitation of the validation study was the lack of available data for a more robust examination of reliability beyond internal consistency, such as test-retest.

**Conclusions:**

The six scales developed by Schieman and Young (2013) hold promise as measures of work contact, work-family conflict, psychological distress, and sleep problems in relation to working conditions of construction professionals.

## Background

In a 2011 study using a telephone survey of 5729 adults in the general working population of Canada, Schieman and Young (2013) [1] investigated the effects of after-hours, work-related contact (‘work contact’) on individual and family health and well-being. Using boundary theory [2] and the Job Demands-Resources (JD-R) model of workplace stress [3] as theoretical frameworks, Schieman and Young (2013) [1] employed regression analysis to investigate whether work-family conflict, psychological distress, and sleep problems are associated with work contact. Their survey design, done in three stages, incorporated eight core antecedent and outcome constructs (job autonomy; schedule control; challenging work; job pressure; work contact; work-family conflict; psychological distress; and sleep problems). In the first instance, work-family conflict was posited as the outcome of the job demand and resource variable antecedents. In the second instance, psychological distress was posited as the outcome of work-family conflict in addition to the job demand and resource variable antecedents. Finally, sleep problems were posited as the outcome of work-family conflict and psychological distress, in addition to the job demand and resource variable antecedents. Support was found for all three hypotheses. It is not fully clear how the study selected individual items for their eight constructs, and no evidence of formal psychometric validation of their scales was presented.

The Schieman and Young (2013) [1] study was replicated by Bowen et al. (2017) [4] using a modified instrument and different method of survey administration to explore similar issues (using path analysis) of work contact among 630 construction professionals in South Africa. Both studies investigated the same question i.e., whether work-family conflict, psychological distress and sleep problems are associated with work contact. As with Schieman and Young (2013) [1], the Bowen et al. (2017) [4] study employed the scales without detailed psychometric evaluation of the items and scales comprising these items.

The aim of the current study, using the same dataset as Bowen et al. (2017) [4], is to assess the construct validity and internal consistency of the modified Schieman and Young (2013) [1] measurement *scales* through confirmatory factor analysis and tests for internal consistency (α). Comprehensive overviews of the theoretical frameworks underpinning the prior research of Schieman and Young (2013) [1] and Bowen et al. (2017) [4] can be found in those publications.

### Survey instrument modification

The Bowen et al. (2017) [4] study modified the Schieman and Young (2013) [1] telephone survey instrument in several respects. The modifications were made to permit the following: application of more robust data analytic techniques; a specific focus on construction professionals as compared to the general population of working adults; and administration of an online rather than a telephonic survey. The structure of the modified instrument is shown in Table 1 and is followed by an explanation of the changes made. Unless otherwise stated, the items, time frames, and response options employed by Schieman and Young (2013) [1] were retained to facilitate the validation of the measurement scales developed by Schieman and Young (2013) [1].

**Table 1:** Scale items for variables and scales

Items	Response options
1. Demographic variables	
Gender	Male = 1; Female = 2
Relationship status	Divorced, separated, widowed or never married = 1; Married or living with a partner = 2
Children under 18 years residing at home	None = 1; 1 Child = 2; 2 Children = 3; 3 Children = 4; 4 Children = 5; 5 Children = 6; 6 Children = 7; 7 Children = 8; Exceeding 7 Children = 9
Experience in the construction industry	1–5 years = 1; 6–10 yrs. = 2; 11–15 yrs. = 3; 16–20 yrs. = 4; Exc. 20 yrs. = 5
Employment position	Salaried employee = 1; Associate = 2; Director or Partner = 3
2. Job Autonomy and Control (JAC) (Scale score range: 3–12)	
JAC1. You have the freedom to decide what you do on your job? [C18a]	Strongly disagree = 1; Somewhat disagree = 2; Somewhat agree = 3; Strongly agree = 4
JAC2. It is your own responsibility to decide how your job gets done? [C18c]	
JAC3. You have a lot to say about what happens on your job? [C18d]	
3. Job pressure (JP) (Scale score range: 3–15)	
In the last 3 months, how often did (were):	
JP1. You feel overwhelmed by how much you had to do at work? [C19a]	Never = 1; Rarely = 2; Sometimes = 3; Often = 4; Very often = 5
JP2.You have to work on too many tasks at the same time? [C19b]	
JP3. The demands of your work exceed the time you have to do the work? [C19c]	
4. Work contact (WC) (Scale score range: 3–15)	
In the past 3 months:	
WC1. How often were you called about work matters outside of normal office hours? [C20a]	Never = 1; Rarely = 2; Sometimes = 3; Often = 4; Very often = 5
WC2. How often did you receive job-related emails or text messages out of normal office hours? [C20b]	
WC3. How often did you contact people about work matters outside of normal office hours? [C20c]	
5. Work-family conflict (WFC) (Scale score range: 4–20)	
In the past 3 months:	
WFC1. How often did you not have sufficient time for important people in your life because of your job? [C21a]	Never = 1; Rarely = 2; Sometimes = 3; Often = 4; Very often = 5
WFC2. How often did you not have sufficient energy to do things with important people in your life because of your job? [C21b]	
WFC3. How often did your work keep you from doing as good a job at home as you could? [C21c]	
WFC4. How often did your job keep you from concentrating on important things in your family or personal life? [C21d]	
6. Psychological distress (PD) (Scale score range: 7–35)	
In the past month, how often did you (feel):	
PD1. Anxious or tense? [B3a]	None of the time = 1; A little of the time = 2; Some of the time = 3; Most of the time = 4; All of the time = 5
PD2. Nervous? [B3b]	
PD3. Worry a lot about things? [B4a]	
PD4. Have you had trouble keeping your mind on what you were doing? [B4b]	
PD5. Feel restless or fidgety? [B4c]	
PD6. Sad or depressed? [B5a]	
PD7. Hopeless? [B5c]	
7. Sleep problems (SP) (Scale score range: 3–15)	
In the past month how often have you:	
SP1. Had trouble falling or staying asleep? [B6a]	None of the time = 1; A little of the time = 2; Some of the time = 3; Most of the time = 4; All of the time = 5
SP2. Woke up before you wanted to? [B6b]	
SP3. Woke up feeling refreshed? *(R)* [B6c]	

Note: (*R*) indicates item was reverse coded. Question number references are given in parentheses

Three items were used to assess the extent of job autonomy and control experienced by respondents. These were retained as is from the Schieman and Young (2013) [1] instrument and are based on similar items used in the National Study of the Changing Workforce (see [5]). The 4-point response options used followed Galinsky et al. (2001) [6], but no time frame was specified in either instance.

The Schieman and Young (2013) [1] items for *challenging work* (demand) and *schedule control* (control) were omitted. The *challenging work* variable was excluded on the grounds that all construction-related professional work should be regarded as skilled and challenging in nature; whereas Schieman and Young (2013) [1] were exploring general work situations where that might not be so, i.e., workers engaged in menial or boring tasks. The *schedule control* variable is not wholly relevant to this study as construction professionals (such as architects, engineers and quantity surveyors) typically enjoy considerable control in relation to the timing of their work and are rarely compelled to work specific hours (e.g., compared to a shift worker on a production line). This variable was deemed to be subsumed into the *job autonomy and control* variable.

For the *job pressure* variable, the three items used by Schieman and Young (2013) [1] were retained. They are based on the earlier work of Karasek et al. (1998) [7], Carayon and Zijlastra (1999) [8], Galinsky et al. (2001) [6], Kristensen et al. (2004) [9], and Harma (2006) [10] and explore respondents’ perceptions of job pressure experienced in the previous 3 months. No evidence could be found of any one source from which Schieman and Young (2013) [1] drew their questions. Rather, they used items similar to previously used items on similar themes documented in the literature indicated above. The response options and time frame used by Schieman and Young (2013) [1] followed Galinsky et al. (2001) [6].

The three items measuring the frequency of survey participants’ *work contact* experiences in the preceding 3 months were retained. Schieman and Young (2013) [1] are silent regarding the source of their questions, possible response options, and time frame. Notwithstanding, the Schieman and Young (2013) [1] items were kept in order to facilitate the psychometric validation of these scales.

The *work-family conflict* variable was retained. It comprises the four items drawn from Schieman and Young (2013) [1], and previously employed by Voydanoff (2007) [11] and Schieman and Young (2010a, 2010b) [12, 13] to assess the extent of work-family conflict experienced in the preceding 3 months. The time frame and response options align with those used by Voydanoff (2004, 2005) [14, 15], but no explanation is provided by Schieman and Young (2013) [1] for the choice of these specific items.

For the *psychological distress* variable, the seven items used by Schieman and Young (2013) [1] were retained. These were drawn from the Kessler K10 index of generalised psychological distress (Kessler et al., 2002) [16] and assess self-reported psychological distress as experienced by survey respondents in the preceding month. Schieman and Young (2013) [1] adopted both the time frame and the response options advocated by Kessler et al. (2002) [16] but did not explain the choice of these seven specific items.

Similarly, the *sleep problems* scale was retained intact. Its three items were drawn from the 10 items employed by Maume et al. (2009) [17] and use a participant self-assessment approach to reporting sleep problems. The time frame adopted by Schieman and Young (2013) [1] was 1 month, as opposed to the 3 months used by Maume et al. (2009) [17]. No explanation is given by Schieman and Young (2013) [1] for the adoption of a different time frame. Moreover, the Maume et al. (2009) [17] study employed four response options (i.e., *never, rarely, sometimes,* or *often*) compared to the five response options used by Schieman and Young (2013) [1] (see Table 1). No explanation is given for the use of different response options, nor for the choice of these three specific items. Following Maume et al. (2009) [17], question SP3 was reverse-worded without explanation from Schieman and Young (2013) [1].

In addition to the questions relating to the six constructs of interest, various demographic characteristics were captured. Participants reported their gender, relationship status, the number of children younger than 18 years old residing at home, years of experience in the construction industry, and employment position. Employment position was categorised as *salaried employee*, *associate*, or *partner or director*. The response options for these demographic variables are shown in Table 1.

### Hypotheses derived from the Schieman and Young (2013) [1] and Bowen et al. (2017) [4] studies

#### Items and scales

Firstly, it was hypothesized that there are 23 items and 6 separate scales covering the constructs of job autonomy and control (3 items), job pressure (3 items), work contact (3 items), work-family conflict (4 items), psychological distress (7 items), and sleep problems (3 items) (Hypothesis 1). Moreover, it was hypothesized that each scale is unidimensional, with each item loading uniquely onto one scale (Hypothesis 2). It was also hypothesized that the items loading onto each scale are as depicted in Table 1 (Hypothesis 3).

#### Inter-relationship between scales

Based on the correlation analysis of scales undertaken by Schieman and Young (2013) [1] (correlation coefficients and significance values shown in parentheses), these authors found positive associations between work-family conflict and each of job pressure (*r* = .54, *n* = 5729, *p* < .01), work contact (*r* = .34, *n* = 5729, *p* < .01), psychological distress (*r* = .41, *n* = 5729, *p* < .01), and sleep problems (*r* = .33, *n* = 5729, *p* < .01). Negative associations were found between work-family conflict and job autonomy (*r* = −.13, *n* = 5729, *p* < .01). In other words, workers experiencing more frequent work contact would also tend to experience more psychological distress and sleep problems. The resource hypothesis predicted that these associations would be weaker among workers enjoying greater job-related resources such as higher levels of job autonomy and control. In contrast, the demand hypothesis indicated that those positive associations would be stronger among workers experiencing more job pressure.

Ordinary least squares (OLS) regression techniques were used by Schieman and Young (2013) [1] to test their predictions. Specifically, the regression of work-family conflict on (inter alia) work contact, job pressure, and job autonomy; and the regressions of each of psychological distress and sleep problems on (inter alia) work contact, job pressure, job autonomy and control, and work-family conflict supported their predictions based on the correlation analysis – both in terms of strength and direction of the association.

Whereas Schieman and Young (2013) [1] used regression analysis to develop and test *separate* predictive models for explaining work-family conflict, psychological distress and sleep problems, the Bowen et al. (2017) [4] study specified and tested a number of path models to examine the direct and indirect determinants of psychological distress and sleep problems. *Separate* path models were initially specified and tested, followed by an integrated, composite path model predicting both psychological distress and sleep problems. The path coefficients derived from the composite model indicated that work-family conflict was positively associated with job pressure (*β* = .50, *p* < .001) and work contact (*β* = .22, *p* < .001), but negatively associated with job autonomy and control (*β* = −.11, *p* < .001). Psychological distress was positively associated with job pressure (*β* = .29, *p* < .001) and work-family conflict (*β* = .32, *p* < .001), and sleep problems were positively associated with work contact (*β* = .07, *p* < .05), work-family conflict (*β* = .11, *p* < .01), and psychological distress (*β* = .55, *p* < .001). Finally, job pressure was found to be negatively associated with job autonomy and control (*β* = −.13, *p* < .01). It was therefore hypothesized that these same relationships would be found in the present study (Hypothesis 4). The Bowen et al. (2017) [4] study did not present correlation analysis of the associations between the six constructs, hence its inclusion in the present paper and incorporation into Hypothesis 4.

#### Internal consistency (Cronbach’s alpha)

Schieman and Young (2013) [1] (*n* = 5729) and Bowen et al. (2017) [4] (*n* = 630) found the constructs under consideration to reflect the alpha values presented hereafter. The Bowen et al. (2017) [4] values are presented first: job autonomy and control (3-items) (0.83; .78); job pressure (3-items) (.91; .85); work contact (3-items (.84; .78); work-family conflict (4-items) (.91; .90); psychological distress (7-items) (.90; .83); and sleep problems (3-items) (.75; .72).

In both of the studies cited above the respective alpha values were provided without discussion of item-total correlations, the values of alpha if an item was deleted, nor the influence of test length (see de Vet et al., 2017 [18]: Spearman-Brown prophecy formula), and sample size. The present study addresses these issues. Additionally, the alpha values reported by Bowen et al. (2017) [4] were derived from the usable dataset (*n* = 630). In the current study, this same dataset was randomly divided into two discrete sub-samples to facilitate psychometric validation. Assessments of internal consistency were undertaken separately for each sub-sample (explained more fully below).

It was hypothesized that the alpha values generated in the present study would fall within the ranges outlined above (Hypothesis 5), noting however, that alpha values are affected by a number of factors, namely, the correlation between the items, the length of the scale, the width of the scale, and the sample size. If the items in a scale are highly correlated, the value of alpha is increased. In terms of the Spearman-Brown prophecy formula [18], if the test length is short, the value of alpha is reduced. Larger sample sizes can lead to higher alpha values [19, 20]. Items in a scale with fewer response options are associated with lower alpha values [21]. These aspects will be explored during the assessment of the internal consistency of the scales under review.

#### Convergent validity

For the convergent validity analysis of the final scales, the following items drawn from Schieman and Young (2013) [1] were utilised: industrial experience, feeling annoyed or frustrated at work, being preoccupied with work matters when not at work, and co-worker support to manage work and family responsibilities. The choice of these particular items is justified further on.

Specifically, it was hypothesized that industrial experience would be positively associated with both job autonomy and control and work contact, and negatively associated with job pressure, work family conflict, psychological distress and sleep problems (Hypothesis 6). Thus, it was predicted that construction professionals with more experience would report higher levels of job autonomy and control and work contact, and lower levels of job pressure, work-family conflict, psychological distress, and sleep problems. More experienced professionals were more likely to be in senior positions within firms, enjoying greater job autonomy and control, and would be more likely to have developed coping mechanisms for dealing with job pressure (and resultant psychological distress and sleep problems).

It was hypothesized that feeling frustrated or annoyed at work would be positively associated with job pressure, work contact, work-family conflict, psychological distress and sleep problems, and negatively associated with job autonomy and control (Hypothesis 7). That is, professionals enjoying greater job autonomy would be more likely to experience less frustration at work, but professionals exposed to high levels of job pressure, work contact, and work-family conflict would be more likely to feel frustration at work. The latter group would also be more likely to experience psychological distress and sleep problems than the former group.

It was hypothesized that preoccupation with work matters when not actually at work would be positively associated with job autonomy and control, job pressure, work contact, work-family conflict, psychological distress, and sleep problems (Hypothesis 8). In essence, construction professionals experiencing higher levels of work preoccupation, and hence less emotional detachment from the work environment, would be more likely to experience higher levels of job autonomy and control, job pressure, work contact, work-family conflict, psychological distress and sleep problems than would their less work-preoccupied colleagues.

Finally, it was hypothesized that co-worker support would be positively associated with job autonomy and control, but negatively associated with job pressure, work contact, work-family conflict, psychological distress, and sleep problems (Hypothesis 9). It was predicted that professionals experiencing higher levels of support from co-workers, assistance that helps them balance work and family life, would be more likely to enjoy greater job autonomy and control, and lower levels of pressure, work contact, work-family conflict, psychological distress, and sleep problems, than would construction professionals who experience lower levels of co-worker support.

## Methods

### Primary data collection from the population

The population consisted solely of professionals employed in the construction industry in South Africa: that is, architects, engineers, quantity surveyors, and project and construction managers registered with their respective statutory councils. Professional registration is mandatory in South Africa, so registration / membership lists are excellent proxies for full populations. Registered professionals were emailed by their respective statutory bodies (assisted where necessary by the voluntary professional institutions), provided with a URL for online access to the questionnaire, and asked to participate in the survey. No inducement was offered to participants.

Of the 942 responses received, 78 returns were substantially incomplete and were removed. The reduced dataset (*n* = 864) represented 9% of the total professional population in the country, comprising 297 (35%) architects, 294 (34%) engineers, 184 (21%) quantity surveyors, and 89 (10%) project and construction managers. Since many in the lattermost group hold dual registration in another discipline, their actual representation in the response sample is likely to be higher.

### Statistical analysis

Using IBM SPSS [22] a variety of descriptive and bivariate statistical analyses was performed. To verify the factorial structure of all measured variables, confirmatory factor analysis (CFA) using structural equation modeling (SEM) was performed on both sub-samples A and B. The sub-sample sizes were deemed sufficient for CFA [23]. Modification indices, available within AMOS (see below), were used to guide the model revision process.

CFA, using maximum likelihood estimation to evaluate model fit, was conducted on the sub-samples using IBM AMOS Version 24.0 for Windows [24]. Given the categorical nature of the data, Bayesian estimation (the methodological approach available within AMOS for analyzing categorical data using the *Markov Chain Monte Carlo* (MCMC) *algorithm*) was used to compare the parameter estimates derived from both the ML and Bayesian approaches. Specifically, in Bayesian estimation, the mean of the posterior distribution can be reported as the parameter estimate (regression weight) and the standard deviation of the posterior distribution serves as an analog to the standard error in ML estimation [25]. In this instance, Bayesian estimation was used to verify the parameter estimates derived from the ML estimation approach [25]. Sampling convergence was deemed to have occurred when the convergence statistic was less than 1.002 [26]. Byrne (2010: 160) [25] contends that “…. *where the hypothesized model is well specified and the scaling based on more than three categories, it seems unlikely that there will be much of a difference between the findings”.*

Five critical model fit indices were applied to determine the degree of fit of the structural equation models, as follows (indices reflecting good model fit indicated in parenthesis): χ^2^/*df* ratio (less than 4); Bollen’s IFI (incremental fit index (.95 and greater); Bentler CFI (comparative fit index (.95 and greater)); RMSEA (root mean square error of approximation (.06 and less)); and Hoelter (critical N (CN) index) (200 and greater) [27]. RMSEA is regarded as the most informative statistic in determining model fit as it takes cognizance of the number of variables being estimated in the model [25]. Model improvements were tested using the Chi-Square Difference Test [28]. Once the factorial structure had been validated, unweighted scale scores were created by summating the scores of their respective constituent items with reverse scoring of individual items where appropriate.

After model structure was confirmed, the convergent validity of the model and internal consistency of the scales were assessed. Convergent validity at scale level was assessed using four variables drawn from the Schieman and Young (2013) [1] study. These variables were selected on the basis of their face validity [29] i.e., when the content of the research is related to the studied variables in terms of logic. The selected variables comprised: extent of ‘*industrial (work) experience’*, ‘*feeling angry, and frustrated, at work in the last month*’ (‘none of the time’; ‘a little of the time’; ‘some of the time’; ‘most of the time’; and ‘all of the time’); ‘*thinking about work-related issues when not working*’ (‘never’; ‘rarely’; ‘sometimes’; ‘often’; ‘very often’); and ‘*I have the support from co-workers that helps me to manage my work and personal or family life*’ (‘strongly disagree’; ‘somewhat disagree’; ‘somewhat agree’; ‘strongly agree’; and ‘not applicable’). With the exception of industrial experience, these additional items are not shown in Table 1.

If the relationships between the rationalization scores and the empirically-related variables discussed above are confirmed, then the test of the convergent validity of the scale would be satisfied [30, 31]. Pearson correlation coefficients (for continuous variables) were used to assess convergent validity.

Cronbach’s alpha was calculated to assess the internal consistency of each scale. Higher value means better internal consistency and Cronbach’s alpha >.7 is considered acceptable [31].

## Results

### Missing values and data analysis

The reduced dataset (*n* = 864) was subjected to across-scale and scale-wise missing value analysis using Little’s MCAR test. Missing values were indicated for items on all six scales. In terms of missing values, 22 out of 23 items, 109 out of 864 cases, and 222 out of 19,872 values indicated missing values. Four (i.e., nervous, fidgety, tense, and hopeless) of the seven items measuring psychological distress indicated the greatest number of missing values.

Little’s MCAR test indicated that the assumption that all item missing values were missing completely at random was tenable as follows: *job autonomy and control* (χ^2^ = 5.03, *df* = 6, *p* = .54, range .6–.7%); *job pressure* (χ^2^ = .35, *df* = 2, *p* = .84, range .7–.9%); work contact (χ^2^ = 1.04, *df* = 2, *p* = .59, range .7–.8%); work-family conflict (χ^2^ = 4.71, *df* = 5, *p* = .45, range .8–1.4%); psychological distress (χ^2^ = 128.23, *df* = 111, *p* = .13, range .7–3.8%); and sleep problems (χ^2^ = 1.31, *df* = 4, *p* = .86, range 0–.8%).

Little’s MCAR test was also performed on all items across all measures simultaneously (χ^2^ = 860.53, *df* = 829, *p* = .22, range 0–3.8%), again indicative that item missing values were missing completely at random. The extent of missing values was low for all six measures (less than 4%), and accordingly listwise deletion was adopted [32].

This resulted in a 630-case reduced dataset (hereafter termed the ‘final dataset’) with no missing values. The demographic characteristics in this final dataset were almost identical to that in the dataset of 864 cases.

The final dataset was randomly divided into two discrete sub-samples. This is the recommended protocol for psychometric validation in a single sample [33]. The randomised split was performed using IBM SPSS (Statistical Package for the Social Sciences) Version 24.0 for Macintosh [22]. Sub-sample A contained 311 cases, whilst the second sub-sample B contained 319 cases. The characteristics of both sub-samples are shown in Table 2. Analysis confirmed that both sub-samples were equivalent in terms of demographics and other key variables, except for the *work contact* variable. Significantly (at a 95% confidence interval) higher levels of work contact were indicated in sub-sample A (*M* = 9.66, *SD* = 2.81) than in sub-sample B (*M* = 9.15, *SD* = 2.83; *t* (628) = 2.25, *p* = .03). However, the magnitude of the difference in the means (mean difference = .51, 95% *CI*: .06 to .95 was very small (*eta* squared = .008) and may be disregarded [34].

**Table 2:** Characteristics of participants in the randomly split sub-samples A (*n* = 311) and B (*n* = 319)

Characteristics	Sub-sample A (*n* = 311)		Sub-sample B (*n* = 319)	
	*n*	%	*n*	%
Demographic characteristics				
Gender^b^				
Male	262	84	255	80
Female	49	16	64	20
Relationship status^b^				
Divorced, separated, widowed or never married	37	12	42	13
Married or living with a partner	274	88	277	87
Children under 18 years residing at home				
None	156	50	165	52
1 Child	50	16	40	13
2 Children	90	29	97	30
3 Children	12	4	14	4
4 Children	1	0.4	3	1
5 Children	1	0.3	0	0
6 Children	0	0	0	0
7 Children	1	0.3	0	0
Exceeding 7 Children	0	0	0	0
Experience in the construction industry^a^				
1–5 years	17	5	20	6
6–10 years	34	11	37	12
11–15 years	38	12	40	12
16–20 years	43	14	44	14
Exc. 20 years	179	58	178	56
Employment position^a^				
Salaried employee	101	32	98	31
Associate	34	11	30	9
Director or partner	176	57	191	60
	Mean	SD	Mean	SD
Behavioural characteristics				
Job autonomy and control (JAC)^c^				
JAC score (Range 3–12)	9.74	2.21	9.77	2.01
Job pressure (JP)^c^				
JP score (Range 3–15)	11.01	2.85	11.04	2.77
Work contact (WC)^c,d^				
WC score (Range 3–15)	9.66	2.81	9.15	2.83
Work-family conflict (WFC)^c^				
WFC score (Range 4–20)	12.70	3.74	12.61	3.74
Psychological distress (PD)^c^				
PD score (Range 7–35)	17.46	5.73	17.42	5.54
Sleep problems (SP)^c^				
SP score (Range 3–15)	8.72	2.87	8.33	2.73

Notes: ^a^ The Chi-square test for independence or the ^b^ Fisher’s Exact Test was used for categorical variables, and the ^c^ independent samples t-test was used for continuous variables. ^d^ No differences were found between sub-sample characteristics and means, except for *work contact*; with sub-sample A depicting significantly higher levels of work contact than did sub-sample B

### Demographic characteristics of the sample

Using the final dataset (*n* = 630), the age of participants ranged from below 25 years to over 60 years, with the mean and median ages in the interval 45–49 years. Most participants were male (82%), and either married or living with a partner (88%). Just under half (49%) reported children under 18 years old living at home, and most had either one (14%) or two (30%) children.

The mean duration of experience in the construction industry was 16–20 years, whilst the median was greater than 20 years. Just over half of respondents were partners or directors (58%), associates comprised 10%, and about a third were salaried employees (32%).

Almost a third of respondents (32%) reported typically working in excess of 50 h per week, with 16% working 56 h or more per week. Just over a quarter (26%) claimed to work (on average) more than 10 h per week at home on job-related work, and 8% reported working in excess of 30 h per week in this way.

To investigate possible bias due to self-selection, we attempted to compare the distribution of the survey respondents and the whole population regarding socio-demographic characteristics i.e., gender, ethnicity, age, and employment position. Information on age and employment position was unavailable. Regarding gender, official statistics put the proportion of women at 50% of all economically-active professionals in the South African economy [35], but the percentage of professional women in the construction sector is significantly lower. According to the Council for the Built Environment (2013) [36], the representation of professional women is as follows: architects (19%), engineers (3%), quantity surveyors (15%) and project and construction managers (3%). Of the 630 responses, females accounted for 18% of the total and 34% of architects, 6% of engineers, 20% of quantity surveyors and 8% of project and construction managers. Women were thus overrepresented in all professional groupings in the current study.

The Council for the Built Environment (2013) [36] reported that “Whites” account for 73% of architects, 77% of engineers, 74% of quantity surveyors and 82% of project and construction managers. Of the 630 respondents to this survey, 89% were “White” (93% of architects, 93% of engineers, 81% of quantity surveyors and 80% of project and construction managers) and the remainder were “Black”, “Coloured” or “Indian”. Thus, “White” respondents were slightly over-represented in the current study. The ethnic categories used in this study refer to demographic markers and do not denote any inherent characteristics. Their continued use in post-*apartheid* South Africa is considered important for monitoring and assessing improvements in diversity compliance in employment practices in all sectors, including the construction industry. The issue of possible self-selection and cultural bias is explored more fully under Limitations.

### Scale scores

Participants’ scores on the summated *job autonomy and control* scale ranged from 3 to 12, with a mean of 9.76 (*SD* = 2.11) and a median of 10. The summated scale scores for the other scales were as follows: *job pressure* (Range: 3–15, *M* = 11.02, *SD* = 2.81; *Md* = 11); *work contact* (Range: 3–15, *M* = 9.40, *SD* = 2.83; *Md* = 9); *work-family conflict* (Range: 4–20, *M* = 12.66, *SD* = 3.74; *Md* = 12); *psychological distress* (Range: 7–34, *M* = 17.44, *SD* = 5.63; *Md* = 17); and *sleep problems* (Range: 3–15, *M* = 8.52, *SD* = 2.81; *Md* = 8). In all instances, higher scores indicate higher levels of the attribute of interest. Full details pertaining to the sub-samples are given in Table 2.

### Analysis of sub-sample A (*n* = 311)

#### Correlation analysis using sub-sample A

Table 3 reports the correlations (one-tailed tests) between the six constructs of interest using sub-sample A. All of the correlations were significant (one-tailed). Specifically, all were correlated *p* < .001 with the exception for the correlation between job autonomy and control and job pressure, work contact, work-family conflict, and sleep problems (see Table 3).

**Table 3:** Correlations between the six scales (sub-sample A) (*n* = 311)

Constructs	JAC	JP	WC	WFC	PD	SP
1. Job autonomy and control (JAC)	–					
2. Job pressure (JP)	−.15**	–				
3. Work contact (WC)	.14**	.43***	–			
4. Work-family conflict (WFC)	−.16**	.64***	.46***	–		
5. Psychological distress (PD)	−.26***	.47***	.26***	.50***	–	
6. Sleep problems (SP)	−.15**	.34***	.25***	.42***	.63***	–

Notes: Score ranges: JAC (3–12), JP (3–15), WC (3–15), WFC (4–20), PD (7–35), and SP (3–15)

*p < 0.05; **p < 0.01; ***p < 0.001 (one-tailed test)

The *magnitude* of the relationships varied between small (.10 to .29), medium (.30 to .49) and large (≥ .50) [34]. Notably, the strength of the relationships between job autonomy and control and the other constructs was small (.10 to .29), as was the case of the relationships between work contact and both psychological distress and sleep problems. The strength of the association between work contact and work-family conflict, and between work-family conflict and sleep problems was medium. Notably, the strength of the relationship between work-family conflict and psychological distress, and between psychological distress and sleep problems, was strong.

The directions of the relationships were positive in all instances except in relation to job autonomy and control. Specifically, increased job autonomy and control was associated with lower levels of job pressure, work-family conflict, psychological distress and sleep problems, respectively. Increased job autonomy and contact was positively associated with work contact.

In terms of shared variance (*r*^*2*^), job pressure explained 41.0% of the variance in work-family conflict and 22.1% of the variance in psychological distress, respectively. Work contact explained 21.2% of the variance in work-family conflict, and work-family conflict explained 25.0% and 17.6% of the variance in psychological distress and sleep problems, respectively. Notably, psychological distress explained 39.7% of the variance in sleep problems.

The significance, strength and direction of these correlations align closely to those reported by Schieman and Young (2013) [1].

#### Confirmatory factor analysis using sub-sample A

Confirmatory factor analysis was first conducted on sub-sample A (*n* = 311). A measurement model based on the six factors adapted from Schieman and Young (2013) [1] was specified and tested. Output indices for the CFA model indicated a satisfactory fit to the data (χ2/*d*f ratio = 1.91, IFI = .96, CFI = .96, RMSEA = .05, and Hoelter (95%) = 190). All factor loadings were statistically significant (*p* < .001). The modification indices indicated the need for a correlated error: PD4 (‘*difficulty in concentrating*’) with PD5 (‘*fidgety*’). Correlated errors should not be permitted atheoretically simply to improve model fit, but rather on the basis of reasoned justification. However, error term correlation per se should not be avoided *de rigueur*, as not to do so may result in model fit that will possibly be slanted in a conservative manner to some extent [37].

With this correlated error specified, the resultant model presented a very good fit to the data (χ2 /*df* ratio = 1.77, IFI = .96, CFI = .96, RMSEA = .05, and Hoelter (95%) = 204), with all factor loadings statistically significant (*p* < .001). The Chi-Square Difference Test revealed that this model was a significant improvement on the previous model [(Δχ^2^(1) = 30.43, *p* < .001), indicating that the inclusion of this correlated error substantively enhanced the model. No further modifications were considered necessary.

Bayesian estimation was employed to compare the parameter estimates for this model derived from both the ML and Bayesian approaches. The convergence statistic cutpoint was 1.0015. The results are depicted in Table 4. The parameter estimates were very close to each other. Inspection of the Bayesian SEM diagnostic first and last combined polygon plot for each item indicated that AMOS successfully identified salient features of the posterior distribution for each item. The Bayesian SEM diagnostic trace plots indicated convergence in distribution occurred rapidly, a clear indicator that the SEM model was specified correctly [25].

**Table 4:** Comparison of the factor loadings (regression weights), depicting the unstandardized parameter estimates for the measurement models derived from sub-samples A and B using the Maximum Likelihood versus the Bayesian estimation methods

Factors and items	Measurement Model: Sub-sample A		Measurement Model: Sub-sample B	
	Maximum Likelihood Estimation (std. err.)	Bayesian estimation (std. dev.)	Maximum Likelihood Estimation (std. err.)	Bayesian estimation (std. dev.)
JAC1 ← Job autonomy and control	1.000	1.000	1.000	1.000
JAC2 ← Job autonomy and control	.865 (.062)	.860 (.062)	.837 (.071)	.825 (.070)
JAC3 ← Job autonomy and control	.912 (.064)	.908 (.066)	1.011 (.083)	.998 (.085)
JP1 ← Job pressure	1.000	1.000	1.000	1.000
JP2 ← Job pressure	.961 (.049)	.958 (.051)	.895 (.041)	.894 (.042)
JP3 ← Job pressure	1.094 (.053)	1.089 (.056)	.984 (.045)	.980 (.047)
WC1 ← Work contact	1.000	1.000	1.000	1.000
WC2 ← Work contact	1.063 (.077)	1.049 (.079)	1.126 (.082)	1.117 (.088)
WC3 ← Work contact	1.071 (.079)	1.058 (.082)	.928 (.074)	.921 (.074)
WFC1 ← Work-family conflict	1.000	1.000	1.000	1.000
WFC2 ← Work-family conflict	1.113 (.068)	1.106 (.065)	1.209 (.085)	1.200 (.080)
WFC3 ← Work-family conflict	1.182 (.066)	1.177 (.068)	1.270 (.083)	1.254 (.078)
WFC4 ← Work-family conflict	1.126 (.066)	1.118 (.066)	1.239 (.081)	1.227 (.079)
PD1 ← Psychological distress	1.000	1.000	1.000	1.000
PD2 ← Psychological distress	.965 (.058)	.967 (.057)	.950 (.054)	.949 (.054)
PD3 ← Psychological distress	.994 (.064)	.996 (.065)	.927 (.057)	.928 (.057)
PD4 ← Psychological distress	.771 (.063)	.772 (.064)	.754 (.062)	.754 (.063)
PD5 ← Psychological distress	.987 (.062)	.987 (.062)	.953 (.061)	.954 (.062)
PD6 ← Psychological distress	.804 (.057)	.807 (.058)	.850 (.057)	.849 (.060)
PD7 ← Psychological distress	.881 (.063)	.885 (.065)	.812 (.059)	.814 (.060)
SP1 ← Sleep problems	1.000	1.000	1.000	1.000
SP2 ← Sleep problems	.864 (.072)	.864 (.065)	.879 (.074)	.873 (.070)
SP3 ← Sleep problems	.730 (.067)	.730 (.075)	.603 (.066)	.601 (.072)

The lowest inter-factor correlation indicated by the model was between job autonomy and control and work contact (*r* = .16), aligning with the Pearson’s product moment correlation (*r* = .14; *p* < .05) (see Table 3).

#### Internal consistency using sub-sample A

Using sub-sample A, the internal consistency of the six scales confirmed in the CFA was assessed using Cronbach’s alpha. The analysis indicated satisfactory-to-good internal consistency: job autonomy and control (3-items) (α = .85), job pressure (3-items) (α = .91), work contact (3-items) (α = .84), work-family conflict (4-items) (α = .91), psychological distress (7-items) (α = .91), and sleep problems (3-items) (α = .77). These alpha values align closely with those reported by Schieman and Young (2013) [1] and Bowen et al. (2017) [4]. For all six scales the corrected item-total correlation values exceeded .50 (indicative of very good discrimination), with the exception of item SP3 (‘*woke up feeling refreshed*’ [reversed]), which was .499.

None of the six scales indicated an improvement in internal consistency occasioned by the removal of one or more items from that scale, with the notable exception of the sleep problems scale. Specifically, the removal of SP3 (‘*woke up not feeling refreshed*’) from the scale would raise the alpha value of the scale from .77 to .79. The deletion of item SP3 from the scale would result in a 2-item scale, a situation cautioned against [31].

The length of these scales should be taken into consideration when interpreting the above results. To increase alpha, more related items testing the same concept should be added to the test, but that was not possible in this study.

### Analysis of sub-sample B (*n* = 319)

#### Confirmatory factor analysis using sub-sample B

To test the robustness of the measurement model developed using sub-sample A (*n* = 311), CFA was performed using sub-sample B (*n* = 319). Initially, no correlated errors were permitted. Output indices for the CFA model indicated a satisfactory fit to the data (χ2/*d*f ratio = 2.49, IFI = .93, CFI = .93, RMSEA = .07, and Hoelter (95%) = 149), although all factor loadings were statistically significant (*p* < .001). The modification indices indicated the need for a correlated error: PD4 (‘*difficulty in concentrating*’) with PD5 (‘*fidgety*’).

Regarding the inclusion of the correlated errors between PD4 (‘*difficulty in concentrating*’) with PD5 (‘*fidgety*’), and in the context of the holdout sample, the correlation coefficients of the seven items measuring psychological distress ranged from .40 to .73. All were significant, *p* < .001. Items PD4 and PD5 were the most highly-correlated items (*r* = .73), indicating 53.3% shared variance and large magnitude.

**Fig. 1:**
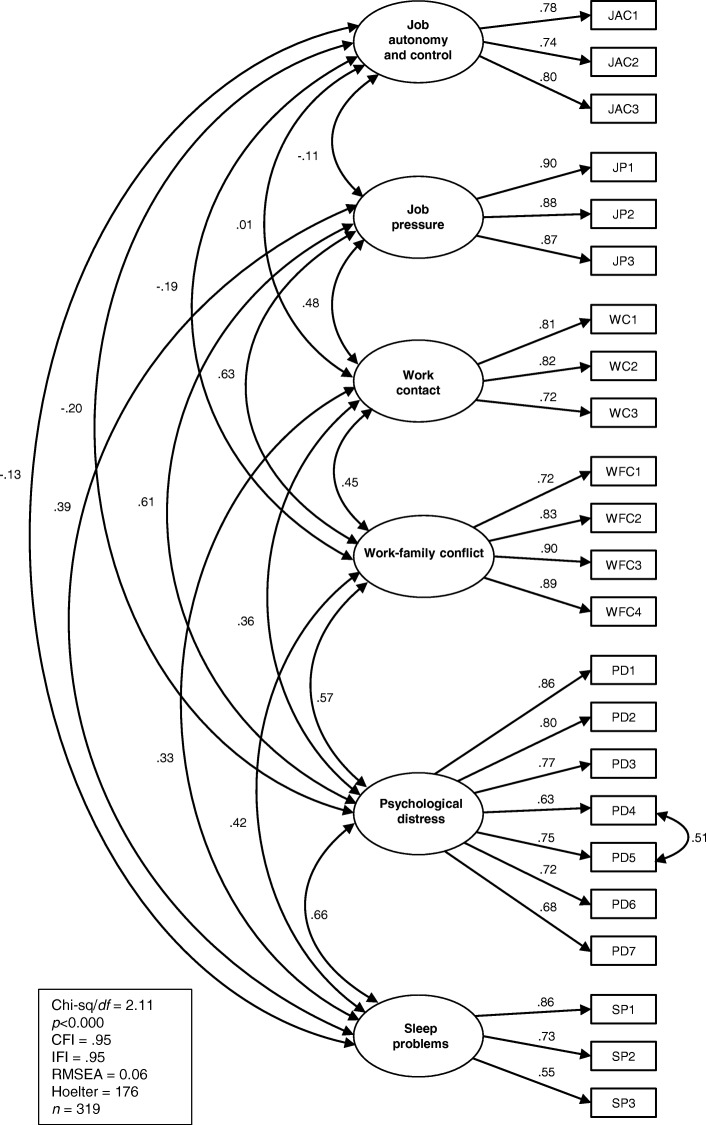
Confirmatory factor analysis of the 6-factor model

After the inclusion of this correlated error, the resultant model presented a very good fit to the data (χ2 /*df* ratio = 2.11, IFI = .95, CFI = .95, RMSEA = .06, and Hoelter (95%) = 176), with all factor loadings statistically significant (*p* < .001) (see Fig. 1). The Chi-Square Difference Test revealed that this model was a significant improvement on the previous model [(Δχ^2^(1) = 83.23, *p* < .001), indicating that the inclusion of this correlated error substantively enhanced the model. No further modifications were necessary. The issue of correlated error terms is considered more fully in the Discussion section. The Hoelter value of 176 is below the recommended threshold of 200 [27] but is most likely a function of sample size.

In this CFA the lowest inter-factor correlation was again between job autonomy and control and work contact (*r* = .01) (aligning with the Pearson’s product moment correlation, *r* = .002; *p* = .49), in contrast with the lowest inter-factor correlation indicated in the CFA of sub-sample A (JAC-WC; *r* = .16). The association between work contact and job autonomy and control is the only aspect in which the models for sub-samples A and B diverge. A possible reason for this anomaly is contemplated in the Discussion section. Exclusion of this relationship from the model does not significantly improve model fit to the data [(Δχ^2^(1) = .04, *p* > .05) and the relationship was therefore retained.

Again, Bayesian estimation was employed to compare the parameter estimates for this model derived from both the ML and Bayesian approaches. The convergence statistic cutpoint was 1.0017. The results are depicted in Table 4. The parameter estimates are very close to each other. Inspection of the Bayesian SEM diagnostic first and last combined polygon plot for each item also indicated that AMOS successfully identified salient features of the posterior distribution for each item. Similarly, the Bayesian SEM diagnostic trace plots spoke well for the validity of our hypothesized structure of the scales.

The final model is depicted in Fig. 1.

#### Internal consistency using sub-sample B

Using sub-sample B (*n* = 319), the internal consistency of the six scales confirmed in the CFA was assessed using Cronbach’s alpha. The analysis indicated satisfactory-to-good internal consistency: job autonomy and control (3-items) (α = .82), job pressure (3-items) (α = .91), work contact (3-items) (α = .83), work-family conflict (4-items) (α = .90), psychological distress (7-items) (α = .90), and sleep problems (3-items) (α = .73). These alpha values again align closely with those reported by Schieman and Young (2013) [1] and Bowen et al. (2017) [4].

None of the six scales indicated an improvement in internal consistency as a result of the removal of one or more items from that scale, again with the notable exception of the sleep problems scale. The removal of SP3 (‘*woke up not feeling refreshed*’) [reversed] from the scale would raise the alpha value of the scale from .73 to .80, but would result in a 2-item scale [31].

The corrected item-total correlation values for all six scales exceeded .50 (indicative of very good discrimination), with the exception of item SP3 (‘*woke up feeling refreshed*’), which was .41 and indicative of poor discrimination. For the psychological distress scale, item PD4 (‘*difficulty in concentrating*’) indicated the lowest corrected item-total correlation, *r* = .64.

#### Convergent validity of the scales using sub-sample B

The CFA indicated that the final measurement model described above is a very good fit to the data. The model demonstrates construct validity at an item level. This model comprises 23 items with a 6-factor structure. As outlined above, four variables were used to assess convergent validity, namely, work experience, frustration at work, work pre-occupation, and co-worker support. Results of the validity tests at a scale level are described below. The relevant correlation coefficients (one-tailed tests), together with their respective significance and strength of the relationships, are depicted in Table 5.

**Table 5:** Correlations between selected variables and the six scales (sub-sample B: *n* = 319)

Constructs	Work experience	Frustration at work	Work pre-occupation	Co-worker support at work
1. Job autonomy and control (JAC)	.39***	−.20***	.03	.24***
2. Job pressure (JP)	−.15**	.37***	.44***	−.31***
3. Work contact (WC)	.14**	.25***	.41***	−.15**
4. Work-family conflict (WFC)	.00	.43***	.52***	−.45***
5. Psychological distress (PD)	−.19***	.64***	.41***	−.36***
6. Sleep problems (SP)	−.03	.52***	.39***	−.28***

Notes: *p < 0.05; **p < 0.01; ***p < 0.001 (one-tailed test). The magnitude of the relationships is defined as follows:

trivial (*r* < .10), small (*r* = .10 to .29), medium (*r* = .30 to.49), and large (*r* ≥ .50)

Work experience was significantly correlated with all factors except with *work-family conflict* and *sleep problems* (inversely). Specifically, work experience was positively associated with *job autonomy and control* (*r* = .39, *n* = 319, *p* < .001) and *work contact* (*r* = .14, *n* = 319, *p* < .01), but inversely associated with *job pressure* (*r* = −.15, *n* = 319, *p* < .01), and *psychological distress* (*r* = −.19, *n* = 319, *p* < .001). Thus, construction professionals with more work experience reported higher levels of job autonomy and control and work contact, but lower levels of job pressure and psychological distress. Whilst the relationships described above are significant, their magnitude is small, except between work experience and job autonomy and control where it is medium. The effect of work experience on work-family conflict and sleep problems was trivial (<.10) [34].

Feeling frustrated or annoyed at work was significantly inversely related to *job autonomy and control* (*r* = −.20, *n* = 319, *p* < .001), and significantly positively related to *job pressure* (*r* = .37, *n* = 319, *p* < .001), *work contact* (*r* = .25, *n* = 319, *p* < .001), *work-family conflict* (*r* = .43, *n* = 319, *p* < .001), *psychological distress* (*r* = .64, *n* = 319, *p* < .001), and *sleep problems* (*r* = .52, *n* = 319, *p* < .001). Whilst significant, the magnitude of the association between frustration at work and each scale is as follows: small (job autonomy and control, and work contact); medium (job pressure and work-family conflict); and large (psychological distress and sleep problems). Notably, frustration at work explains 41.0% and 27.0% of the variance in respondents’ scores on the psychological distress and sleep problem scales, respectively.

Preoccupation with work matters when not actually at work was significantly positively associated with *job pressure* (*r* = .44, *n* = 319, *p* < .001), *work contact* (*r* = .41, *n* = 319, *p* < .001), *work-family conflict* (*r* = .52, *n* = 319, *p* < .001), *psychological distress* (*r* = .41, *n* = 319, *p* < .001), and *sleep problems* (*r* = .39, *n* = 319, *p* < .001). For all scales except job autonomy and control and work-family conflict, the magnitude of association with work pre-occupation is medium. With job autonomy and control it is trivial, whilst with work-family conflict it is large. Pre-occupation with work explains 27.0% of the variance in respondents’ scores on the work-family conflict scale.

Co-worker support was significantly positively associated with *job autonomy and control* (*r* = .24, *n* = 275, *p* < .001), and significantly negatively associated with *job pressure* (*r* = −.31, *n* = 275, *p* < .001), *work contact* (*r* = −.15, *n* = 275, *p* < .01), *work-family conflict* (*r* = −.45, *n* = 275, *p* < .001), *psychological distress* (*r* = −.36, *n* = 275, *p* < .001), and *sleep problems* (*r* = −.28, *n* = 275, *p* < .001). The magnitude of the association between co-worker support and each scale is as follows: small (job autonomy and control, work contact, and sleep problems); and medium (job pressure, work-family conflict, and psychological distress).

## Discussion

The aim of our study was to examine the psychometric properties of the scales offered by Schieman and Young (2013) [1] in their regression-based examination of the determinants of psychological distress and sleep problems in relation to work contact.

The CFA using the holdout sample (*n* = 319) verified the factorial structure of the set of 23 variables loading onto a 6-factor structure, confirming the hypothesized relationships between the observed variables and their underlying latent constructs.

The psychological distress scale contained seven items and five response options. Scale length exceeded that of all the other scales. High correlation between items, longer tests, and greater response options are associated with higher internal consistency (alpha) [20]. This scale demonstrated good internal consistency, and the removal of any item from this scale would not improve internal consistency. However, item PD4 (‘*difficulty in concentrating*’) indicated the lowest corrected item-total correlation. It is contended that the high correlation between PD4 and PD5, together with the low corrected item-total correlation associated with PD4, resulted in the need to correlate the error terms of these items in both measurement models. Permitting correlated errors between PD4 and PD5 resulted in very good model fit to the data in both sub-samples. Support for Hypothesis 1 was therefore indicated, subject to the reservation presented above. Bayesian estimation proved a suitable substitute for the maximum likelihood approach to parameter estimation. All scales were found to be unidimensional, with all items loading strongly onto their respective scales. Thus, support for Hypotheses 2 and 3 was also indicated.

In terms of the inter-relationships between the scales, work-family conflict was positively associated with job pressure and work contact, but negatively associated with job autonomy and control. Psychological distress was positively associated with job pressure and work-family conflict, and sleep problems were positively associated with work contact, work-family conflict, and psychological distress. Finally, job pressure was found to be negatively associated with job autonomy and control. These results variously supported Hypothesis 4.

The final model, replicated in sub-sample B (see Fig. 1), indicated a trivial correlation between the two factors job autonomy and control and work contact. This was not the case with sub-sample A, and therefore the relationship was retained. A possible reason for this anomaly may lie in the fact that, notwithstanding the two sub-samples being equivalent in terms of all of the other variables of interest, a significantly higher level of work contact was indicated in sub-sample A than in sub-sample B.

In terms of internal consistency, the scales were found to be internally consistent to a satisfactory-to-good degree, aligning closely with those reported by Schieman and Young (2013) [1] and Bowen et al. (2017) [4].

One scale item proved problematic in terms of both obtained alpha value and corrected item-total correlation, namely, item SP3 (‘*woke up feeling refreshed*’ [reversed]). This item was the only one in the questionnaire to have been reversed-worded. Schieman and Young (2013) [1] are silent on the possible effects of this reverse-worded item on the efficacy of the sleep problems scale. Reverse-worded items are employed in an attempt to reduce or prevent response bias. Research indicates that using reverse-worded items can result in scores being contaminated by respondent inattention and confusion [38, 39]. It is quite possible for some respondents to have misinterpreted the direction of the response options associated with this particular item, resulting in measurement error and distorted scores. The use of items posed in the same direction is advocated, noting that such a format is preferable for both epidemiological and clinical studies [38, 39].

Removal of item SP3 from the sleep problems scale would have slightly increased the alpha value, but removal of this item from the scale would have resulted in a 2-item scale [31]. Future research would usefully be directed at incorporating a more suitable item.

Although these results need to be interpreted with caution, considering the length of the scales the Cronbach’s alphas can be considered satisfactory and therefore Hypothesis 5 was considered supported.

The convergent validity analysis indicated that the scales were generally associated with professional’s work experience, feeling annoyed or frustrated at work, being preoccupied with work matters when not at work, and co-worker support, in terms of *direction*. However, there was no association between professionals’ work experience and the work-family conflict and the sleep problems they experience, respectively. Similarly, there was no association between the extent of professionals’ job autonomy and control and their preoccupation with work matters when not at work. These results were not anticipated. A possible reason for the lack of association between work-family conflict and professionals’ work experience may lie in the nature of work undertaken by construction professionals. Specifically, construction professionals, irrespective of seniority or years of experience, are expected to be self-motivated, work independently, and assume responsibility for the projects (or parts thereof) assigned to them. The job pressures generated by such responsibilities, coupled with associated after-hours contact, conceivably give rise to work-family conflict notwithstanding differences in work experience.

The lack of association between sleep problems and work experience is less clear. Given the negative relationship between work experience and psychological distress, albeit small, and the strong positive association between psychological distress and sleep problems, a negative relationship between work experience and sleep problems could have been anticipated. Specifically, it would have been reasonable to assume that older, more experienced professionals would have developed coping mechanisms to deal with workplace stress, thereby experiencing less psychological distress and sleep problems than would their younger, less experienced colleagues. This anomaly warrants more detailed examination in future research.

The lack of association between professionals’ job autonomy and control scale and their after-hours work pre-occupation may be explained by the same reasons as outlined above for the lack of association between work experience and work-family conflict. In essence, irrespective of job autonomy and control (itself positively related to work experience and seniority), construction professionals demonstrated a preoccupation with work matters when not physically at work.

Regarding the magnitude of the associations, none of the six scales indicated *strong* (large) associations with the items in question - with the notable exception of the relationships between each of the psychological distress and sleep problem scales with feeling frustrated at work; and the association between the work-family conflict scale and after-hours preoccupation with matters relating to work. The remaining associations were of medium magnitude at best. With the exception of the job autonomy and control scale (medium magnitude), the associations between work experience and the remaining scales were small to trivial. A possible reason for this may lie in the pressurized nature of the work undertaken by construction professionals, regardless of status within a firm and extent of experience.

Thus, Hypotheses 6, 7, 8 and 9, were largely supported with respect to direction of association, but not fully supported in relation to magnitude of association.

Overall, the results confirmed the model structure, the underlying construct validity, and the internal consistency of the six scales. Convergent validity was largely demonstrated with respect to direction of association, but not in relation to magnitude.

### Limitations

This study has several limitations. The sector-specific nature of the study limits generalization to the general population. However, given the similarity of construction industries globally, our findings would likely be applicable beyond the South African construction industry. Moreover, it is also likely that this study would be generalizable to professional consultants operating in other economic sectors which share similar job pressures and work environment demands with respect to time, schedule flexibility and frequency of work contact (e.g., the medical professions), but this would need to be investigated further.

We indicated the self-selecting and self-reporting nature of the survey to highlight the voluntary nature of participation, cautioning that such voluntary participation might reflect individuals with very strong views either way about work contact and the causes and effects thereof. We acknowledge the potential bias inherent in this as a limitation of the study, and that there can be no absolute claim to *complete* representivity. However, we do not believe respondent bias is a serious concern.

Regarding cultural bias, notwithstanding the fact that the vast majority of respondents were “White” (89%), exceeding the ethnicity statistic relating to each professional group provided by the Council for the Built Environment, there is no reason to believe that cultural practices and traditions would bias the results to any great degree within the context of the professional practice of built environment practitioners. It is acknowledged that age may be a differentiating factor in any assessment of cultural bias, but such an investigation was beyond the scope of this study.

The survey was cross-sectional in nature, hence test-retest reliability could not be investigated. Similarly, given the small number of items per scale, split-half reliability could not be assessed. While internal consistency is recognized as an important aspect of scalar psychometric integrity, it is acknowledged that the test for scale *reliability* is incomplete without at least another form of reliability testing, preferably test-retest reliability.

Finally, to facilitate the psychometric validation of the scales developed by Schieman and Young (2013) [1], we adopted the time frames that they used, acknowledging the different time frames used for the different scales. Schieman and Young (2013) [1] are not forthcoming on reasons for the choice of different time frames.

Despite these limitations, and subject to the caveats raised above, it is argued that the scales evaluated here do represent promising empirical measures of job autonomy and control, job pressure, work contact, work-family conflict, psychological distress, and sleep problems as experienced by South African construction professionals.

## Conclusions

This study examined the psychometric properties of the job autonomy and control, job pressure, work contact, work-family conflict, psychological distress, and sleep problem scales developed by Schieman and Young (2013) [1]. Survey data were obtained from 942 construction professionals in South Africa, of which responses from 630 respondents were considered for analysis. The scales demonstrated very good model fit and all factor loadings were significant. There was supportive evidence indicating the internal consistency of the scales, but evidence confirming the convergent validity of the scales was more equivocal. Convergent validity was largely demonstrated with respect to direction of association, but not in relation to magnitude.

Given the similarity of construction processes, and to some considerable extent building procurement processes, in construction industries worldwide, it is possible that the scales (subject to any necessary specific contextual modification, such as language) could be applied in other contexts for exploring work-related stress issues among construction professionals. The extent of application and relevance to such contexts would logically be contingent upon the degree of modification necessary in terms of issues such as language of administration, and mode of survey administration.

## Abbreviations

AMOS: Analysis of Moment Structures
CFA: Confirmatory Factor Analysis
JD-R: Job Demands-Resources model of workplace stress
SEM: Structural Equation Modelling
SPSS: Statistical Package for the Social Sciences
URL: Uniform Resource Locator
